# Voltage-Gated Sodium Channels: Biophysics, Pharmacology, and Related Channelopathies

**DOI:** 10.3389/fphar.2012.00124

**Published:** 2012-07-11

**Authors:** Eleonora Savio-Galimberti, Michael H. Gollob, Dawood Darbar

**Affiliations:** ^1^Division of Cardiovascular Medicine, Department of Medicine, Vanderbilt UniversityNashville, TN, USA; ^2^Division of Cardiology, Department of Medicine and Cellular and Molecular Medicine, University of OttawaOttawa, ON, Canada

**Keywords:** voltage-gated sodium channels, channelopathies, electrophysiology, sodium channels, pharmacology, biophysics

## Abstract

Voltage-gated sodium channels (VGSC) are multi-molecular protein complexes expressed in both excitable and non-excitable cells. They are primarily formed by a pore-forming multi-spanning integral membrane glycoprotein (α-subunit) that can be associated with one or more regulatory β-subunits. The latter are single-span integral membrane proteins that modulate the sodium current (*I*_Na_) and can also function as cell adhesion molecules. *In vitro* some of the cell-adhesive functions of the β-subunits may play important physiological roles independently of the α-subunits. Other endogenous regulatory proteins named “*channel partners”* or “*channel interacting proteins*” (ChiPs) like caveolin-3 and calmodulin/calmodulin kinase II (CaMKII) can also interact and modulate the expression and/or function of VGSC. In addition to their physiological roles in cell excitability and cell adhesion, VGSC are the site of action of toxins (like tetrodotoxin and saxitoxin), and pharmacologic agents (like antiarrhythmic drugs, local anesthetics, antiepileptic drugs, and newly developed analgesics). Mutations in genes that encode α- and/or β-subunits as well as the ChiPs can affect the structure and biophysical properties of VGSC, leading to the development of diseases termed sodium “*channelopathies*”.  This review will outline the structure, function, and biophysical properties of VGSC as well as their pharmacology and associated channelopathies and highlight some of the recent advances in this field.

## Introduction

In mammals, 11 genes (*SCN1A–SCN11A*) encode a family of nine functionally expressed voltage-gated sodium channels (VGSC; Na_v_1.1–Na_v_1.9) that share more than 50% amino acid sequence homology (Catterall et al., 2005). α-subunits encoded by these genes are organized into four homologous domains (DI–DIV), each one of which is composed of six transmembrane segments. Segments 1 through 4 of each domain form the voltage sensor, while segments 5 and 6 (and their connecting linker the P-loop) compose the pore region. In addition to the α-subunit, VGSC also include β-subunits (which are mainly regulatory molecules) as integral parts of the channel. VGSC can interact with other endogenous proteins called “*channel partners*” or “*channel interactive proteins*” (ChiPs) that modulate channel expression and/or function. The last group currently includes caveolin-3 (Lu et al., 1999; Yarbrough et al., 2002; Vatta et al., 2006; Cronk et al., 2007), calmodulin/calmodulin kinase II (CaMKII; Maier and Bers, 2002; Tan et al., 2002; Wagner et al., 2006; Pitt, 2007), connexin-43 (Sato et al., 2011; Chkourko et al., 2012), telethonin (Mazzone et al., 2008), plakophilin (Sato et al., 2009), ankyrins (Kordeli et al., 1995, 1998; Davis et al., 1996; Garrido et al., 2003; Mohler, 2006), neuronal precursor cell-expressed developmentally down regulated 4 (nedd4; Ingham et al., 2004; Rougier et al., 2005), fibroblast growth factor homologous factors (FHFs; Liu et al., 2001, 2003b; Laezza et al., 2007; Dover et al., 2010; Wang et al., 2011; Goldfarb, 2012), membrane-associated guanylate kinase synapse-associated proteins (SAPs; Petitprez et al., 2011; Milstein et al., 2012), and the syntrophin/dystrophin complex (Haenggi and Fritschy, 2006; Shao et al., 2009).

Mutations in the genes encoding the VGSC have been associated with a wide variety of diseases including Dravet syndrome and other types of epilepsy (Claes et al., 2001; Mantegazza et al., 2005; Mullen and Scheffer, 2009), pain-related syndromes [which includes congenital insensitivity to pain (CIP), primary erythromelalgia (PE), and paroxysmal extreme pain disorder (PEPD) (Dib-hajj et al., 2009; Lampert et al., 2010)], and cardiac arrhythmias [which includes congenital long QT syndrome (LQTS) type 3 (Wang et al., 1995), Brugada Syndrome (BS; Probst et al., 2003), progressive cardiac conduction defect (Scott et al., 1999), sick sinus syndrome (Benson et al., 2003), atrial fibrillation (AF; Olson et al., 2005; Darbar et al., 2008), slow ventricular conduction (Chambers et al., 2010; Sotoodehnia et al., 2010), and atrial stand still (Tan, 2006; Remme et al., 2008)]. Epigenetic up regulation of VGSC has recently been associated with aggressive metastatic carcinoma of prostate (Na_v_1.7) and breast (Na_v_1.5) (Onkal and Djamgoz, 2009). The up regulation of the VGSC seems to occur early in the dissemination of this type of cancer and ignite the metastatic status (the VGSC expression positively correlated *in vivo* with invasiveness and therefore metastatic spread) (Onkal and Djamgoz, 2009). Because of their central role in the pathophysiology of these diseases, VGSC are clear pharmacological targets as sites of action for antiepileptic drugs, newly developed analgesics and antiarrhythmic drugs, and potential disease markers in metastatic carcinomas (prostate, breast).

This review will focus on the structure, function, and biophysics of the VGSC, as well as their pharmacology, the sodium channel “*partners*” (or “ChiPs”) currently identified and the sodium “*channelopathies*”.

## Structure of VGSC

Voltage-gated sodium channels are heteromeric integral membrane glycoproteins that can be differentiated by their primary structure, kinetics, and relative sensitivity to the neurotoxin tetrodotoxin (TTX). They are composed of an α-subunit of approximately 260 kDa (~2000 amino acids), that is associated with one or more regulatory β-subunits (β1–β4) of approximately 35 kDa each (Catterall, 2000). We will describe in detail both subunits (α and β) that conform the VGSC.

### α-subunits

Ten different mammalian α-subunit isoforms (Na_V_1.1–Na_V_1.9 and Na_X_) have been characterized (Table 1) and at least seven of them are expressed in the nervous system. Na_V_1.1, Na_V_1.2, Na_V_1.3, and Na_V_1.6 isoforms are mainly expressed in the central nervous system (CNS). In contrast, Na_V_1.7, Na_V_1.8, and Na_V_1.9 isoforms are predominantly located in the peripheral nervous system (PNS; Ogata and Ohishi, 2002), are known to accumulate in the region of peripheral nerve injury and may be important in chronic, neuropathic pain (Devor, 2006; Table 1). In recent reports *SCN10A*/Na_V_1.8 has also been identified in human hearts (Facer et al., 2011; Yang et al., 2012) and in intracardiac neurons (Verkerk et al., 2012), where genetic variations in the *SCN10A* gene have been associated with alterations in the PR interval, QRS duration, and ventricular conduction (Chambers et al., 2010; Sotoodehnia et al., 2010). Because these isoforms (Na_V_1.1–1.3, Na_V_1.6–1.9) are mainly localized in nervous tissue they are generally referred as “*brain type*” or “*neuronal-type*” sodium channels. Na_V_1.4 isoform is mainly expressed in skeletal muscle, while Na_V_1.5 is the cardiac-specific isoform. The isoform referred to as “Na_X_ channel” [also named NaG/SCL11 (rats), Nav2.3 (mice), and/or hNav2.1 (humans)] identifies a subfamily of sodium channel-like proteins (George et al., 1992). This channel has significant differences in the amino acid sequence in the voltage sensor, inactivation gate, and pore region when compared to the rest of VGSC (George et al., 1992; Goldin et al., 2000). Na_X_ is normally expressed in a variety of organs including the heart, skeletal muscle, uterus, dorsal root ganglia (DRG), and brain [mainly in the circumventricular organs (CVOs)]. The difficulties in the characterization of the biophysical properties of this channel are mainly due to lack of success in expressing the functional protein in heterologous expression systems. Hiyama et al. (2002) generated a mouse model in which the Na_X_ gene was knocked out. This group confirmed that Na_x_ channel was expressed in neurons in the CVOs that play a fundamental role controlling body fluid and ionic balance. This group reported that under thirst conditions, mice lacking Na_x_ showed hyperactivity of the neurons in these areas and ingested excessive salt, while wild-type mice did not. This led the investigators to propose that Na_X_ was involved in the mechanism that senses sodium levels in the brain, where this protein might sense extracellular sodium concentration (Hiyama et al., 2002; Noda, 2006).

**Table 1:** **Summary of the different types of VGSC, and the channelopathies associated to mutations in the genes encoding the α subunits**.

Gene	Chromosome	Channel	Expression	TTX	EC50	Human channelopathies
*SCN1A*	2q24.3	Na_V_1.1	Cell bodies of central neurons (“Brain type I”), T-tubules in myocytes (Brette and Orchard, 2006)	S	6 nM (Clare et al., 2000)	Epilepsy and epileptic disorders, including febrile epilepsy and GEFS+ (generalized epilepsy with febrile seizure) (Escayg et al., 2000; Spampanato et al., 2001), Dravet syndrome [severe myoclonic epilepsy of infancy (SMEI)], Doose syndrome (myoclonic astatic epilepsy), intractable childhood epilepsy with generalized tonic-clonic seizures, infantile spasms (West syndrome), Rasmussen’s encephalitis, and Lennox–Gastaut syndrome Non-epileptic disorders: familial hemiplegic migraine (FHM), familial autism, Panayiotopoulos syndrome (Lossin, 2009)
*SCN2A*	2q24.3	Na_V_1.2	Central neurons (“Brain type II”), mainly localized to unmyelinated and premyelinated axons	S	12 nM (Noda et al., 1986)	Inherited febrile seizures and epilepsy (Sugawara et al., 2001)
*SCN3A*	2q24.3	Na_V_1.3	Cell bodies of central neurons (primarily expressed in embryonic/early prenatal life), cardiac myocytes	S	4 nM (Meadows et al., 2002)	Potential contributor to peripheral neuropathic pain after spinal cord injury (Hains et al., 2003)
*SCN4A*	11 (human), 17q23.3 (mouse)	Na_V_1.4	Skeletal muscle (high levels in adult muscle, low levels in neonatal muscle)	S (non-selective)	5 nM (rat; Trimmer et al., 1989), 25 nM (human; Chahine et al., 1994)	Muscle sodium channelopathies (hyperkalemic periodic paralysis, paramyotonia congenital, and potassium-aggravated myotonia, myasthenic syndrome, hypokalemic periodic paralysis type 2, malignant hyperthermia susceptibility; Cannon, 1997)
*SCN5A*	3p21–24	Na_V_1.5	Cardiac myocytes, immature and denervated skeletal muscle, certain brain neurons	R	2–6 μM (Goldin, 2001)	Cardiac sodium channelopathies: Congenital long QT syndrome (Wang et al., 1995; Chen et al., 1998), Idiopathic ventricular fibrillation (Brugada syndrome; Chen et al., 1998; Akai et al., 2000), Isolated cardiac conduction system disease, atrial standstill, congenital sick sinus syndrome, sudden infant death syndrome, dilated cardiomyopathy, other conduction disorders and arrhythmias (George, 2005)
*SCN8A*	15 (human), 12q13 (mouse)	Na_V_1.6	Somatodendritic distribution in output neurons of cerebellum, cerebral cortex, hippocampus; Purkinje cells in cerebellar granule cell layer; astrocytes, and Schwann cells; DRG; nodes of Ranvier in PNS and CNS; T-tubules in cardiac myocytes	S (non-selective)	1 nM (rat; Dietrich et al., 1998), 6 nM (mouse; Smith et al., 1998)	Cerebellar ataxia in jolting mice (Kohrman et al., 1996); motor end-plate disease in mice (Burgess et al., 1995)
*SCN9A*	2q24	Na_V_1.7	All types of DRG neurons, sympathetic neurons, Schwann cells, neuroendocrine cells	S (non-selective)	4 nM (rat), 25 nM (human; Catterall et al., 2005)	Congenital insensitivity to pain (CIP), familial primary erythromelalgia, and paroxysmal extreme pain disorder (PEPD; Lampert et al., 2010)
*SCN10A*	3p22.2	Na_V_1.8	DRG neurons, human heart (Facer et al., 2011; Yang et al., 2012), and intracardiac neurons (Verkerk et al., 2012)	R	60 mM (Catterall et al., 2005)	Peripheral pain syndromes; the channel is up regulated in some models of inflammatory pain; alterations in PR interval and ventricular conduction in the heart (Chambers et al., 2010; Sotoodehnia et al., 2010).
*SCN11A*	3p22.2	Na_V_1.9	c-type neurons in DRG (nociception)	R	40 mM (Catterall et al., 2005)	Potential role in nociception and hyperalgesic syndromes
*SCN7A*	2q24.3	Na_X_	DRG neurons; neurons of hippocampus, thalamus, and cerebellum, median preoptic nucleus, but mainly in the circunventricular organs (CVO); PNS; heart; skeletal muscle; uterus	Unknown	–	Potential role in temporal lobe epilepsy (Gorter et al., 2010); the lack of Na_X_ in neurons from CVO would affect the ability to control body fluids and ionic balance (Hiyama et al., 2002; Noda, 2006)

*S, sensitive; R, resistant; CNS, central nervous system; PNS, peripheral nervous system; DRG, dorsal root ganglia*.

**Figure 1:**
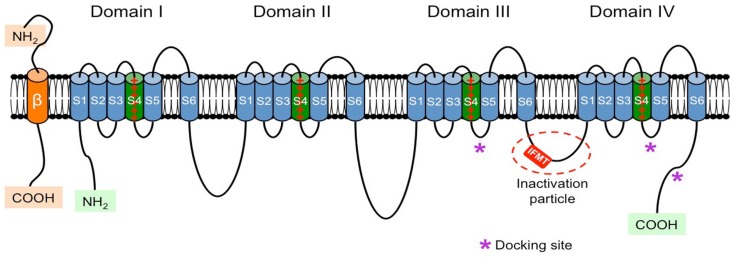
**Schematic representation of the α- and β-subunits of the VGSC**. The four homologous domains (I–IV) of the α-subunit are represented; S5 and S6 are the pore-lining segments and S4 is the core of the voltage sensor. In the cytoplasmic linker between domains III and IV the IFMT (isoleucine, phenylalanine, methionine, and threonine) region is indicated. This is a critical part of the “inactivation particle” (inactivation gate), and substitution of aminoacids in this region can disrupt the inactivation process of the channel. The “docking site” consists of multiple regions that include the cytoplasmic linker between S4–S5 in domains III and IV, and the cytoplasmic end of the S6 segment in domain IV (*). Depending on the subtype of β-subunit considered they could interact (covalently or non-covalently) with the α-subunit.

Each α-subunit is arranged in four homologous domains (DI–DIV) that contain six transmembrane segments (S1–S6; Figure 1). Using cryo-electron microscopy Sato et al. (2001) showed that these four domains are arranged around the central pore of the channel. Segment 4 of each domain contains a high concentration of positive charges (mostly arginine) and functions as the core of the voltage sensor responsible for the voltage-dependent activation of the channels. Segment 6 from all four domains forms the inner surface of the pore. The hairpin-like loop between segments 5 and 6 [S5–S6 hairpin-like P(ore)-loop] is part of the pore of the channel and forms a narrow (ion-selective) filter that controls the ion selectivity and permeation at the extracellular side of the pore (Catterall, 2000; Yu and Catterall, 2003; George, 2005).

Payandeh et al. (2011) recently reported the crystal structure of Na_V_Ab, a VGSC found in the bacterium *Arcobacter butzleri*. Na_V_Ab is part of the NachBac channel family, which is a well-established model to study vertebrate Na_V_ and Ca_V_ channels (Ren et al., 2001; Koishi et al., 2004; Payandeh et al., 2011). Payandeh et al. (2011) were able to capture this channel in the close configuration when the pore was closed with four activated voltage sensors at a resolution limit of 2.7 Å. Payandeh’s work provides the first insight into the structural basis for voltage-dependent gating ion selectivity and drug block in VGSC. The pore consists of an outer tubular vestibule, a selectivity filter, a central cavity (which can lodge partially hydrated sodium ions) and an intracellular activation gate. The helices that constitute the pore are positioned to stabilize cations in the central cavity through helical-dipole interactions (Doyle et al., 1998; Jogini and Roux, 2005). A second P2-helix forms an extracellular funnel and represents a highly conserved element in sodium channels (Payandeh et al., 2011).

Payandeh and coworkers proposed that in Na_V_Ab the ion conduction pathway is electronegative and the selectivity filter (mainly composed of negatively charged glutamate (Glu) side chains) forms the narrowest constriction near the extracellular side of the membrane. There are 4 Glu 177 side chains that form a 6.5-Å × 6.5-Å scaffold with an orifice of approximately 4.6 Å wide. A profuse mesh of amino acid residue interactions, including hydrogen bonds between glutamine from the P-helix and the carbonyl of Glu, stabilizes the selectivity filter. The radius of the pore suggests that hydrated Na^+^ ions can conduct through the channel. Free diffusion then allows the hydrated Na^+^ to enter the central cavity and move through the open activation gate toward the cytoplasm (Payandeh et al., 2011). This permeation pathway contrasts with the selectivity filter in K^+^ channels, which is much narrower. In this case the smaller radius of the pore can only conduct dehydrated K^+^ ions through direct interactions with backbone carbonyls through a long, narrow pore (Morais-Cabral et al., 2001; Ye et al., 2010).

Identification of the primary structure of VGSC led to the development of the “*sliding helix*” (Catterall, 1986b) and the “*helical screw*” (Guy and Seetharamulu, 1986) models (validated by structure-function studies) to better understand how the voltage sensor operates. Both models suggest that positively charged residues in segment 4 within each domain serve as the gating charges moving outward across the membrane as a consequence of membrane depolarization, initiating the activation process (Catterall, 1986a,b; Guy and Seetharamulu, 1986; Catterall et al., 2010). Catterall and coworkers have extensively described these two models. Basically, four to seven residues positively charged within segment 4 would pair negatively charged residues in segments 1, 2, and/or 3. In this configuration, positively charged residues in segment 4 are pulled inward by the electric field of the resting membrane potential which is negative. As depolarization progresses, the change in the polarity of the membrane potential relieve the electrostatic force and the segments 4 move outward allowing each positive charged amino acid in the segment 4 pairs a negatively charged one. As described by Catterall (2010), this outward movement of the gating charges in segments 4 pulls the linker between segments 4 and 5, curves the segment 6 and initiates the opening of the central pore of the channel. The movement of charged particles to activate the sodium conductance (“*gating charges*” or “*gating current*”) was first predicted by Hodgkin and Huxley (Hodgkin and Huxley, 1952; Catterall, 2010), but Armstrong and Bezanilla (1973) were the first ones that measured it in 1973, combining the techniques of internal perfusion, voltage-clamp, and signal average. Using similar techniques, Keynes and Rojas (1973) confirmed the existence of the gating current the same year. Armstrong and Bezanilla (1974) reported additional properties of this current and strong evidence linking it to the gating of the sodium channels the following year.

### β-subunits

These are integral proteins as well, composed of one extracellular domain (ECD, N-terminal domain), one transmembrane domain, and one intracellular domain (C-terminal domain). The β-subunits are expressed in excitable and non-excitable cells within the nervous system and the heart, and there is some evidence suggesting that these proteins can be expressed in the cells even in the absence of the α-subunit (Patino and Isom, 2010; Table 2). One or more regulatory β-subunits (β1–β4) can associate with one α-subunit. An individual α-subunit can be associated with one non-covalently (β1 or β3) and one covalently (β2 or β4) linked β-subunits (Yu and Catterall, 2003; Catterall et al., 2005; Patino and Isom, 2010). The role of β-subunits has been reviewed in detail by Patino and Isom (2010). The authors remark that β-subunits are regulatory proteins that can act both as cell adhesion molecules (CAMs) and modulate the cell surface expression of the VGSC, enhancing sodium channel density and cell excitability. The latter may be a very important mechanism that regulates nociceptor excitability *in vivo* (Lopez-Santiago et al., 2011). β1 association with contactin or neurofascin (NF)-186 also results in increased VGSC cell surface expression (Kazarinova-Noyes et al., 2001; McEwen and Isom, 2004). Furthermore, β1 and β2 are ankyrin-binding proteins. Mice lacking ankyrin exhibit reduced sodium current (*I*_Na_) density and abnormal *I*_Na_ kinetics (Chauhan et al., 2000), suggesting that β-subunits play important roles in the VGSC–ankyrin complex (Patino and Isom, 2010). The interaction between α- and β-subunits may be particularly critical at the nodes of Ranvier of myelinated axons, since mice lacking β1-subunit have reduced numbers of nodes, alterations in the myelination process, and drastically altered contacts between neurons and glial cells (Chen et al., 2004). Even though proteins within nodal regions are localized normally in these mice, association between VGSC and contactin is disrupted. Loss of β1-subunit dependent protein–protein interactions can lead to changes in the structure of the Ranvier nodes and disrupted saltatory conduction (Chen et al., 2004; Davis et al., 2004). Similar to the β1- subunit, β2 can also modulate the expression of the channel at the cell surface and affect *I*_Na_ density (Isom et al., 1995). β2 (and β4) intracellular domains can translocate into the nucleus and enhance *SCN1A* expression, thus functioning as transcriptional regulators of the VGSC α-subunit.

β-subunits are also critical for cellular migration. β1 and β2 mediate migration of fibroblasts (Xiao et al., 1999) and cancer cells (Brackenbury and Isom, 2008), adhesion, and neurite outgrowth (β1 promotes and β2 inhibits this process, while β3 and 4 have no effect; Davis et al., 2004; McEwen et al., 2009). The effects of β-subunits on cell migration, adhesion, and neurite outgrowth also depends on intracellular transduction events like the activation of proto-oncogene tyrosine-protein kinase fyn by β1 to promote neurite (axon and/or dendrite) outgrowth (Brackenbury et al., 2008).

**Table 2:** **Summary of the different types of β subunits associated with the different VGSC, and the related channelopathies associated with the mutations in the genes that encode them (modified from Patino and Isom, 2010)**.

Gene	Chromosome	β subunit	α subunit	Expression	Channelopathies	Model	Reference
*SCN1B*	19q13.1	β1	Na_V_1.1–Na_V_1.7	Central and peripheral neurons, glia, skeletal, and cardiac muscles.	Seizures and epileptic syndromes: febrile seizures, Dravet syndrome, temporal lobe epilepsy	H H,M H	Coward et al. (2001), Audenaert et al. (2003), Chen et al. (2004), Pertin et al. (2005, 2007), Scheffer et al. (2007), O’Malley et al. (2009), Orrico et al. (2009), Patino et al. (2009)
					Traumatic nerve injury	H	
*SCN2B*	11q23	β2	Na_V_1.1, Na_V_1.2, Na_V_1.5–Na_V_1.7	Central and Peripheral neurons, glia, cardiac muscle.	Multiple sclerosis, Neuropathic pain (post-trauma)	M M	Coward et al. (2001), Pertin et al. (2005), Lopez-Santiago et al. (2006), O’Malley et al. (2009)
					Inflammatory pain, traumatic nerve injury	M H
*SCN3B*	11q23.3	β3	Na_V_1.1–Na_V_1.3, Na_V_1.5	Central and peripheral neurons, adrenal gland, kidney	Temporal epilepsy, Traumatic nerve injury	H H	Casula et al. (2004), van Gassen et al. (2009)
*SCN4B*	11q23.3	β4	Na_V_1.1, Na 1.2, Na_V_1.5	Central and peripheral neurons, glia, skeletal and cardiac muscles.	Huntington’s disease	H,M	Oyama et al. (2006)

*H, human; M, mouse*.

## Biophysical Properties of VGSC

When the cell is depolarized, the outward movement of all segments 4 generates a conformational change that opens the pore and thus activates the channel. This allows sodium to flow into the cell down its electrochemical gradient. *I*_Na_ reaches a maximum within milliseconds and then becomes smaller as the sodium channel stops conducting ions and starts closing. The closure of the channel during maintained depolarization is called *inactivation*. The activation and inactivation processes and the coupling between them (in particular, the outward movement of the voltage sensor) are *voltage-dependent*. All the downstream rearrangements are *voltage-independent*. The refractoriness of the cell is related to the inactivated sodium channels, which cannot reopen until they are completely recovered. This mechanism protects the cell and prevents firing during prolonged depolarization (Goldin, 2003).

The time that the channel remains in a specific state (open or close) as well as the rate at which it transitions from one state to the other affects its ionic conductance and thus the shape of the action potential (AP). During the last decade most of the advances in the biophysics of the ion channels has been obtained by expressing the channels in isolated systems (cell lines or lipid bilayers). But the interpretation of this data has been somehow challenging due to the lack of the correct physiological context. The importance of computational biology was first emphasized by Rudy and Silva (2006) by trying to understand the context in which ion channels operate. Still, such theoretical approach in electrophysiology was initiated by Hodgkin and Huxley (1952). Hodgkin and Huxley (1952) were the first that formulated a model of the AP based on the findings of their pioneering work with voltage-clamp techniques showing voltage-dependent changes in ionic conductance in squid axons. The data reported by Hodgkin and Huxley (1952) suggested that the inward flow of Na^+^ was responsible for the rapid initial positive upstroke of the membrane potential, whereas the outward flow of potassium determined the repolarization of the membrane back to resting levels. The mathematical model was designed to determine whether the sodium and potassium currents they identified could in fact generate an AP that was similar in morphology to their AP experimental recordings. The model proposed by Hodgkin and Huxley was reviewed in detail by Rudy and Silva (2006). Rudy and Silva explained that in this model the conductance of sodium and potassium currents was dependent upon the open probability of a series of activation gates. The activation gates in this model correspond to the α-subunits of the VGSC. Each gate can transition from a closed state (open probability = 0) to an open state (open probability = 1) that is independent of the state of the other gates. An ion can only pass through when all the gates (that is, the four α-subunits that constitute the tetramer VGSC) are open (open probability = 1). Rudy and Silva (2006) also pointed out that the original model has several limitations: the model assumes that intracellular concentrations of Na^+^ and K^+^ do not change during the AP, and it does not consider the inactivation of the channel. It also assumes that opening and closing transition rates are independent (Rudy and Silva, 2006). Rudy and Silva proposed that since the VGSC inactivation has a greater probability of occurring when the channel is open, then inactivation is highly dependent on activation. Thus the independent gating of the Hodgkin and Huxley model would not be valid. To improve the accuracy of the mathematical model, Rudy and Silva (2006) suggest considering the dependence of a given transition on the occupancy of different states of the channel. In the case of the VGSC, the model should consider the dependence of the inactivation transition on the probability that the channel occupies the open state. Finally, the authors report that the different states and the dependence of transitions (both voltage-dependent and independent) between states can then be more accurately predicted using Markov-type models (the importance of computational biology to study the integrated electrophysiology of ion channels has been extensively reviewed by Rudy and Silva (2006)).

*Inactivation* is the process by which an open-channel enters a stable non-conducting conformation when the cell membrane depolarizes. The inactivation process includes *fast*, *slow* and *ultra-slow* *inactivation*. In addition, *long-term inactivation FHF-* and β4*-mediated* are processes distinct from slow inactivation (Goldfarb, 2012). In general, while isoforms Na_v_1.1–Na_v_1.4, Na_v_1.6, and Na_v_1.7 have faster inactivation kinetics, Na_v_1.5, Na_v_1.8, and Na_v_1.9 have slower inactivation.

*Fast inactivation* mimics a “*ball-and-chain*” mechanism, where a cytoplasmic segment of the α-subunit of the VGSC (or inactivating particle) occludes the pore by binding to a docking site (Goldin, 2003). Rohl et al. (1999) were the group that first studied the inactivating particle. It consists of a portion of the cytoplasmic linker connecting domains III and IV, with the critical region centering on a 4-amino acid extent consisting of isoleucine (I), phenylalanine (F), methionine (M), and threonine (T) (IFMT; Goldin, 2003). Phenylalanine and threonine directly interact with the docking site and the distance between I- and T-residues correlates with the ability to inactivate the channel (Goldin, 2003). Amino acid substitution within this critical region can disrupt the inactivation of the channel (Kudora et al., 2000; Miyamoto et al., 2001a,b). The docking site includes the cytoplasmic linker connecting segments 4 and 5 in domains III and IV and the cytoplasmic end of segment 6 in domain IV. This “*ball-and-chain*” mechanism is equivalent to the N-type inactivation described for voltage-gated potassium channels (N-type fast inactivation), which involves occlusion of the intracellular mouth of the pore through binding of a short peptide segment from the N-terminal (Rasmusson et al., 1998). *Fast inactivation* is important during AP repolarization, and in some structures like mammalian nodes of Ranvier (which practically lack phasic potassium channels) it is the only repolarizing force besides the leakage current (Ulbricht, 2005).

*Fast inactivation* can be altered by the carboxyl (C)-terminus of the channel. This is due to electrostatic interactions involving the sixth helix in the C-terminus, which can modulate the interaction of the fast inactivating particle with its docking site. The different amino acid composition of the C-terminus explains the differences observed in fast inactivation between the VGSC isoforms (Mantegazza et al., 2001). Motoike et al. (2004) reported that the C-terminus is actually part of the inactivation gate, as it stabilizes the closed state minimizing the reopening of the channel. Mutations in the C-terminus disrupt fast inactivation and can lead to the LQTS type 3 (Goldin, 2003).

*Fast inactivation* can also be modulated by the interaction with β-subunits. The effect and mechanism is dependent on the specific α- and β-subunits involved and the heterologous expression system being used to express the channel. For example, the β1-subunit accelerates the recovery from inactivation of Na_v_1.5 (Zimmer and Benndorf, 2002) and Na_v_1.2 (Chen and Cannon, 1995; McCormick et al., 1998, 1999) and shifts the voltage-dependence of inactivation in the negative direction (Meadows et al., 2002). The β3-subunit has a similar effect on Na_v_1.5, but it increases persistent current through Na_v_1.2 in tsA-201 cells (cell line derived from human embryonic kidney cells; Goldin, 2003). β4-subunits disrupt VGSC inactivation in neurons, working as endogenous open-channel blockers. This subunit has a short cytoplasmic tail that basically blocks the channel in the open state but rapidly dissociates upon membrane repolarization to generate the resurgent current in some neurons like Purkinje cells (Grieco et al., 2005; Goldfarb, 2012).

*Fast inactivation* can be disrupted and transformed into *long-term inactivation* by the interaction of the α-subunit of the channel with a family of cytoplasmic proteins termed *fibroblast growth factor homologous factors* (FHFs) (Goldfarb, 2012). FHFs act as accessory channel subunits. Several FHFs delay fast inactivation by raising the voltage at which fast inactivation occurs. Dover et al. (2010) reported that all A-type FHFs (specially FHF2A and FHF4A) exert a rapid onset of a distinct mode of long-term inactivation of Na_v_1.6 and other VGSC including cardiac Na_V_1.5 (Dover et al., 2010; Goldfarb, 2012). A-type FHFs accomplish long-term inactivation by providing an independent cytoplasmic gating particle that competes with the channel’s intrinsic inactivating particle for blockade of the channel upon membrane depolarization. The authors reproduced this mechanism by injecting a synthetic peptide corresponding to the A-type FHF particle that mimics the long-term inactivation and opposes sustained firing of excitable cells (Dover et al., 2010). β4-mediated channel block and A-FHF-mediated long-term inactivation have a similar physical mechanism. Both processes are mediated by small cytoplasmic particles that interact with the channel after the depolarization has driven the channels into the open state. In both cases, the blocking particles dock at similar sites deep within the cytoplasmic opening of the channel pore. The main difference between the two processes is the rate of particle dissociation, where β4 particle dissociates rapidly and FHF dissociates far more slowly (Goldfarb, 2012).

*Slow inactivation* is a different process that involves conformational changes of the channel leading to rearrangement of the pore. The process also involves segment 4 of domain IV and segments 5 and 6 of domain II (Goldin, 2003). This mechanism is equivalent to the C-type inactivation mechanism described for potassium channels (Rasmusson et al., 1998). *Slow inactivation* may play a role in regulating excitability by, for example, modulating burst discharges. However, this modulation is complicated since slow inactivation depends on both resting membrane potential and the previous history of AP firing (Ulbricht, 2005).

In addition to *fast* and *slow inactivation* there is a third type of inactivation named *ultra-slow inactivation*. This process was described in Na_v_1.4 when the alanine in position 1529 (A1529) is replaced by aspartate (D) in the domain IV P-loop (Goldin, 2003). Binding of the fast inactivating particle inhibits this process. This result demonstrates that there are interactions (mostly, allosteric modulation) among the different inactivation events (Goldin, 2003).

## “Sodium Channel Partners” or “Channel Interactive Proteins”

The current density and gating properties of VGSC can also be modulated by the differential expression of *channel* “*partners*” or ChiPs (Table 3). These terms designate molecules that affect the aggregation, density, function, and regulation of VGSC. Up-to-day, the main identified VGSC partners include caveolin-3 (and the membrane compartment “caveolae”), CaMKII, connexin-43, telethonin, plakophilin, ankyrins, fibroblast growth factor homologous factors (FHFs), nedd4, SAPs, and syntrophin/dystrophin complex.

**Table 3:** **VGSC protein partners**.

Gene	Chromosome	Protein	Expression site and function	Reference
Cav3	3p25.3	Caveolin-3	Scaffolding protein within caveolar membranes. Also involved in VGSC regulation by a mechanism involving the α subunit of the stimulatory G protein (Gα_s_) through the activation of the βARs on the cell surface	Lu et al. (1999), Schwencke et al. (1999), Rybin et al. (2000), Yarbrough et al. (2002)
CALM 2	2p21	Calmodulin	“Calcium-Modulated Protein.” Ca^2+^-binding protein expressed in all eukaryotic cells	Tan et al. (2002)
CAMK 2A	5q32	CaMKII	Part of a family of serine/threonine kinases that mediate many of the second messenger effects of Ca^2+^	Wagner et al. (2006)
GJA1	6q22.31	Connexin-43	Connexins are assembled in groups of six to form hemichannels, or connexons, and two hemichannels then combine to form a gap junction. The connexin gene family is diverse, with 21 identified members in the sequenced human genome	Sato et al. (2011)
TCAP	17q12	Telethonin	Small protein mainly expressed in skeletal muscle that binds to and is phosphorylated by titin kinase and protein kinase D. Both proteins serve as a scaffold to which myofibrils and other muscle related proteins are attached	Valle et al. (1997), Mayans et al. (1998), Mues et al. (1998), Furukawa et al. (2001), Knoll et al. (2002), Haworth et al. (2004), Kojic et al. (2004), Mazzone et al. (2008)
PKP2	12p11	Plakophilin-2	Fundamental component of the cardiac desmosome, structure present in the intercalated disc	Sato et al. (2009)
ANK2	4q25–27	Ankyrin-B (or ankyrin-2)	Cell membrane proteins that link the integral proteins of the membrane to the underlying spectrin-actin cytoskeleton. Mutations in these genes have been related to long QT syndrome type 4 (ANK2) and Brugada like-syndrome (ANK3)	Jenkins and Bennett (2001), Garrido et al. (2003), Lemaillet et al. (2003), Mohler et al. (2004)
ANK3	10q21	Ankyrin-G (or ankyrin-3)	
FGF3	11q13/13.3	FHFs (FGFs)	Family of cytoplasmic proteins termed fibroblast growth factor homologous factors that can delay fast inactivation of VGSC	Dover et al. (2010)
FGF5	4q21/21–21	
FGF6	12q13/13.32	
FGF11	17p13.1	
FGF12	3q28/29	
FGF13	Xq26.3/27.1	
FGF14	13q33.1/34	
Nedd4 Human	15q–15q21.3	Nedd4	Ubiquitin-protein ligases	Rougier et al. (2005)
SNTG 1	8q11.21	Syntrophin	The protein encoded by this gene is a member of the syntrophin family. Syntrophins are cytoplasmic peripheral membrane proteins that typically contain 2 pleckstrin homology (PH) domains, a PDZ domain that bisects the first PH domain, and a C-terminal domain that mediates dystrophin binding. This gene is specifically expressed in the brain	Gavillet et al. (2006), Haenggi and Fritschy (2006), Shao et al. (2009)
DMD	Xp21.2	Dystrophin	Rod-shaped cytoplasmic protein, and a vital part of a protein complex (*costamere* or dystrophin-assoc. prot.s) that connects the cytoskeleton of a muscle fiber to the surrounding extracellular matrix through the cell membrane	
*SCN1B-SCN4B*	19q13.1 (SCN1B) and 11q23 (SCN2B–4B)	β subunits of VGSC	Regulatory subunits of VGSC expressed in CNS, PNS, and heart (see also Tables 1 and 2)	Isom et al. (1994), Kazarinova-Noyes et al. (2001), Chen et al. (2004), McEwen and Isom (2004), Meadows and Isom (2005)

### Caveolae/caveolin-3

Caveolae are sarcolemmal membrane invaginations that have been implicated in cellular trafficking cascades involving the β-adrenergic receptors (β-AR; Schwencke et al., 1999; Rybin et al., 2000). These membrane invaginations also contain scaffolding proteins named “*caveolins*”. Yarbrough et al. (2002) demonstrated both biochemically and functionally that caveolae are involved in VGSC regulation by a mechanism involving the α-subunit of the stimulatory G protein (Gα_s_) through the activation of the β-ARs on the cell surface. Because direct Gα_s_ activation induces an increase in the number of functional channels at the sarcolemma (Lu et al., 1999), they hypothesized that functional channels were recruited from an intracellular store, allowing a faster presentation of channels to the cell surface after β-stimulation. The authors purified the caveolin-3 rich fraction using immunoprecipitation. VGSC and Gα_s_ are colocalized in the Cav3(+)-fraction, suggesting a physical association of both proteins with the caveolar (Cav3-rich) membrane (Rook et al., 2012). They also reported that the increase in *I*_Na_ induced by isoproterenol stimulation (10 μM) in the presence of a protein kinase A (PKA) inhibitor (PKA-independent increase in *I*_Na_) was abolished when an anti-Cav3 antibody was injected into the cytoplasm of the cell through the pipette. This suggests a direct action of the Gα_s_ on the caveolae, resulting in the presentation of caveolar VGSC to the sarcolemma. Palygin et al. (2008) also demonstrated that the histidine residue at position 41 of Gα_s_ (H41) is a critical residue for the functional increase of *I*_Na_ observed.

### Calmodulin/calmodulin kinase II

Tan et al. (2002) demonstrated that calmodulin regulates sodium channel gating through binding to a region of 25 amino acids located at the C-terminus of the intracellular domain. Wagner et al. further studied the downstream signaling through Ca^2+^/CaM-dependent protein kinase II (CaMKIIδ) in heart cells from two heart failure animal models, where expression and activity of CaMKII are increased by twofold to threefold. They demonstrated that calmodulin regulates Na^+^ channel gating in part via CaMKII. Using two cell models of CaMKII overexpression, they concluded that both acute and chronic overexpression of CaMKIIδc significantly shifted voltage-dependence of Na^+^ channel availability by −6 mV, and the shift was Ca^2+^-dependent. CaMKII also enhanced the inactivation of the channel and slowed its recovery from inactivation. These effects were prevented using CaMKII inhibitors (KN93 or AIP). CaMKII over-expression also increased persistent (late) inward *I*_Na_ and the intracellular Na^+^ concentration (also prevented using CaMKII inhibitors). They reported that CaMKII coimmunoprecipitates with and phosphorylates sodium channels. *In vivo* data suggested that CaMKII overexpression mice were more prone to suffer ventricular arrhythmias, particularly monomorphic ventricular tachycardia. The data as a whole supports the hypothesis that CaMKII regulates sodium channel function in myocytes most likely by association with and phosphorylation of the channels (Wagner et al., 2006).

### Telethonin

Telethonin is a small protein (19 kDa) that is mainly expressed in striated muscle (Valle et al., 1997). This protein binds to and is phosphorylated by titin kinase (Mayans et al., 1998) and protein kinase D (Haworth et al., 2004). One of its many functions includes acting as a stretch sensor in the heart (Knoll et al., 2002), linking sarcomeres to K^+^ channel subunits (Furukawa et al., 2001), and interacting with titin (Mues et al., 1998) and ankyrin-2 (also proposed to behave as stress sensors in muscle; Kojic et al., 2004). Mazzone et al. (2008) hypothesized that telethonin may be relevant in tissues different from striated muscle, where it might also behave as a ChiP. After screening 20 unrelated patients with primary intestinal pseudo-obstruction, the authors identified a patient with a heterozygous mutation, R76C, in the telethonin gene by direct DNA sequencing. The mutation is located in the region of telethonin where the protein has been shown to interact with sarcomeric proteins (muscle LIM protein and titin) in the heart. Using immunostaining and immunoprecipitation they demonstrated that telethonin and Na_V_1.5 were colocalized in mouse hearts. They studied the effects of the R67C mutation on the *in vitro* electrophysiology of *SCN5A* expressed in a Human embryonic kidney cell line (HEK)-293. The coexpression of R67C telethonin with *SCN5A* resulted in a leftward shift in the steady-state activation of the sodium channel, leading to increased Na^+^ entry at resting potential (depolarizing effect). The data supports the hypothesis that telethonin acts as a ChiP (Mazzone et al., 2008).

### Plakophilin

Plakophilin-2 (PKP2) is a fundamental component of cardiac desmosomes. This structure is present in the intercalated disk, the site of end-to-end contact between cardiac myocytes, and provides mechanical integrity between adjacent cells. Na_V_1.5 is also highly localized at the intercalated disks. Combining immunochemistry and electrophysiological studies Sato et al. (2009) demonstrated that PKP2 associates with Na_V_1.5 in the same molecular complex, and that the knockout of PKP2 expression produced a decrease in peak current density, a shift in voltage-dependence inactivation, and a prolongation of time-dependence of recovery from inactivation.

### Connexin-43

Connexin-43 peptides are localized at intercalated disks, where they form gap junctions for electrical coupling of adjacent cells. Sato et al. (2011) showed that AnkG, plakophilin, and connexin-43 are associated at the intercalated disks and that this macromolecular complex may interact with clusters of Na_V_1.5 also present in the disk. More recently, Chourko et al. characterized the remodeling of the gap junction (connexin-43) and VGSC in an ovine model of right ventricular pressure overload induced by pulmonary hypertension. The authors reported significant lateralization of connexin-43, which was colocalized with mechanical junction proteins and microtubule-associated proteins EB1 and Kifb5 (these proteins are responsible for the forward trafficking of connexin-43 to the intercalated disk). There was also a significant reduction in the peak *I*_Na_ and in V_1/2_ activation, a slower recovery from inactivation, with no lateralization of the VGSC (Na_V_1.5). The authors then speculate that the difference in the Na_V_1.5 remodeling respect to the connexin-43 could be explained due to the fact that trafficking of Na_V_1.5 might require molecules that cannot redirect the channel to the lateral membrane. In summary, the data reported support the idea of a partnership between these complexes, previously considered to be independent from each other (Chkourko et al., 2012).

### Ankyrins

Ankyrins are a widely expressed family of “adaptor” proteins responsible for the localization of proteins at specialized membrane domains. From all the members that are included in the ankyrin family, ankyrin-G (“G” from “general”) was initially studied in the brain. In neurons, ankyrin-G colocalizes and copurifies with VGSC (Kordeli et al., 1995; Davis et al., 1996; Garrido et al., 2003; Mohler, 2006). Ankyrin-G is important for the clustering of Na_V_1.2 and 1.6 isoforms at the nodes of Ranvier (Jenkins and Bennett, 2001; Garrido et al., 2003) and also colocalizes with VGSC at the neuromuscular junction (Flucher and Daniels, 1989; Kordeli et al., 1998). A role for ankyrin-G for VGSC targeting in cardiac muscle was hypothesized based on the role of this protein in clustering neuronal VGSC (Mohler, 2006). Ankyrin-G binds to a nine residue domain in the DII–DIII loop in the α-subunit of VGSC (Lemaillet et al., 2003). This binding is required for Na_v_1.5 localization in heart cells (Garrido et al., 2003; Mohler et al., 2004). Since ankyrin-G is primarily expressed at the intercalated disk membrane and T-tubules, it colocalizes with Na_v_1.5 at these specific sites (Lemaillet et al., 2003; Mohler et al., 2004; Bennett and Healy, 2008; Lowe et al., 2008). Mutations in either the sodium channel domain at which ankyrin binds, or in ankyrin itself, can affect the channel expression (Mohler et al., 2004).

### Fibroblast growth factor homologous factors

Fibroblast growth factor homologous factors (FHFs) is a family of cytoplasmic proteins that can interact with VGSC and delay fast inactivation by raising the voltage at which fast inactivation occurs. The role of FHFs as VGSC modulators was already discussed under “Biophysical properties of VGSC.”

### Neuronal precursor cell-expressed developmentally down regulated 4

Neuronal precursor cell-expressed developmentally down regulated 4 (Nedd4) is the prototypical protein in a family of E3 ubiquitin. They select specific proteins for conjugation to ubiquitin, which acts as a marker for protein degradation but also in the sorting of proteins at different steps in biosynthetic and endocytic pathways. They are found in the nucleus and at the plasma membrane. Need4-2 refers to a subgroup of ubiquitin-protein ligases that binds the PY motif of Na_v_1.5 and reduces the sodium current (*I*_Na_) in HEK293 cells by promoting its internalization (Rougier et al., 2005). For more details see the review written by Ingham et al. (2004).

### Synapse-associated proteins

Synapse-associated proteins (also called MAGUK, membrane-associated guanylate kinase) are a family of proteins that include Dlg, SAP97/hDlg, SAP90/PSD-95, SAP102, and PSD-93/chapsyn110. They are composed of multiple sites of protein–protein interactions, like the PDZ domains. SAP are localized either to the pre- or postsynaptic sides of excitatory or inhibitory synapses and play a central role in the molecular organization of synapses, like PSD-95, SAP102, and distribution of the NMDA glutamate receptor at the postsynaptic level. One of the family members, SAP97, is also present in epithelial cells and localized at the lateral membrane between cells (Fujita and Kurachi, 2000). At the cardiomyocytes SAP97 colocalized with Na_v_1.5 at the intercalated disks, determining the existence of a second pool of sodium channels in addition to the channels targeted at lateral membranes by the syntrophin-dystrophin complex (Petitprez et al., 2011).

### Syntrophin/dystrophin complex

Syntrophins (α, β, and γ) bind and localize signaling proteins to the plasma membrane (Shao et al., 2009). Syntrophins can also interact with multiple proteins via two pleckstrin homology domains, a PDZ domain and a conserved syntrophin unique region. The PDZ domain can bind to the last three residues of the C-termini intracellular domain of Na_V_1.4 and 1.5 (Haenggi and Fritschy, 2006). The latter can also complex syntrophin and dystrophin (Gavillet et al., 2006). Syntrophin stabilizes the VGSC in the plasma membrane and reduce its internalization (Shao et al., 2009).

### β-subunits

In addition to the modulation of VGSC function (Johnson and Bennett, 2006), β-subunits play critical roles in the intracellular trafficking of α-subunits, regulating the channel expression levels at the plasma membrane and their role in cell adhesion (Isom et al., 1994). *In vitro* data suggests that β-subunits constitute communication links between adjacent cells, extracellular space (via their interaction with tenascin-C and R), cytoskeleton and intracellular signaling mechanisms, and other ion channels. In particular, the β1-subunit seems to be critical for the interaction of the VGSC with other CAMs and cytoskeletal proteins (Kazarinova-Noyes et al., 2001; Chen et al., 2004; McEwen and Isom, 2004). Due to their roles in the interactions with cytoskeletal proteins, CAMs, and other ion channels, Meadows and Isom (2005) proposed that β-subunits should also be considered as molecular scaffolds of the ion conducting pore (α-subunits), therefore critically affecting channel function, subcellular localization and cell surface expression in a cell-specific and subcellular domain-specific manner (see Table 2).

## Pharmacology of VGSC

Voltage-gated sodium channels are the site of action of many toxins and drugs. At least six sites of action for neurotoxins (sites 1–6) and one receptor site for class I antiarrhythmic drugs, local anesthetics and related antiepileptic drugs are known to exist on the VGSC (Cestele and Catterall, 2000). All of them are located on the α-subunit of the channel. Receptor site 1 binds TTX and saxitoxin (Noda et al., 1989; Hille, 2001). This receptor site is formed by amino acid residues in the pore loops and on the extracellular side of them at the outer end of the pore. The sensitivity of the VGSC to TTX segregates them into two groups (Table 1):

1. TTX-sensitive channels (TTX-S; blocked with TTX in the nanomolar concentration range). This group includes Na_v_1.1, Na_V_1.2, Na_v_1.3, Na_v_1.4, Na_v_1.6, and Na_v_1.7 isoforms.
2. TTX-resistant channels (TTX-R; blocked with TTX in the micromolar-millimolar concentration range). This group includes Na_v_1.5, Na_v_1.8, and Na_v_1.9 isoforms.

Biophysical and pharmacological properties of TTX-S and TTX-R Na^+^ channels are different. TTX-R Na^+^ channels can be blocked by inorganic (Co^2+^, Mn^2+^, Ni^2+^, Cd^2+^, Zn^2+^, La^3+^) and organic Ca^2+^ channel blockers. Typically, TTX-R Na^+^ channels show smaller single-channel conductance, slower kinetics, and a more positive current-voltage relation than TTX-sensitive ones. Li and Zhu (2011) recently reported two chimeric peptides of drosotoxin that can block the activity of both TTX-R and TTX-S channels. The authors proposed that this approach of understanding the molecular determinants of toxins affecting VGSC would allow a more rational design of subtype-specific sodium channel blockers.

The overlapping sites of action of antiarrhythmic drugs, local anesthetics and related antiepileptic drugs are located in the inner cavity of the pore of the channel, and they are formed by amino acid residues located in S6 in domains I, III, and IV (Ragsdale et al., 1994, 1996; Hockerman et al., 1997; Catterall, 2000; Yarov-Yarovoy et al., 2001, 2002; Liu et al., 2003a). Drug affinity can be reduced by mutations in critical residues in the pore. Fundamentally, these drugs bind to their corresponding site of action to change the function of the channel (decrease the sodium current density). They can also change the affinity with which the channel binds the drug depending on the functional conformation or state in which the channel is found (rest, active, inactive; Bruton et al., 2011). Most sodium channel-blocking agents block the channel when it is open or inactivated, and have very little or no effect at all while the channel is in the resting state. Thus, with each AP, the drug binds to the VGSC and blocks them, and then dissociates during repolarization, with the consequent loss of blockage.

The dissociation rate is a key determinant of steady-state block of sodium channels. AP frequency and duration, membrane potential level, and the physicochemical properties of the drug will determine the rate of recovery from blockage. When depolarization frequency increases, the rest interval decreases, and so does the time available for drug dissociation as the drug remains attached to the channel for a longer time and consequently the steady-state channel block increases. The increase in depolarization frequency also represents repetitive openings of the pore that increase the access of drugs to the intracellular site of action (use-dependent block; Hille, 1977, 2001). The rate of recovery from blockage also slows as cells are depolarized, as occurs during ischemia. Increased AP duration results in a relative increase in the time the channel remains in the inactive state and this can also increase the block by drugs that mainly bind to sodium channels in the inactivated state such as lidocaine.

Current treatment of neuropathic pain includes tricyclic antidepressants (amitriptyline, nortriptyline), local anesthetics (lidocaine, mexiletine), and antiepileptic drugs (phenytoin, carbamazepine, lamotrigine). These drugs however have low efficacy in terms of pain control and are associated with adverse effects involving the heart and CNS.

## Diseases Associated with VGSC Mutations (“Channelopathies”)

Ten genes (*SCN1A–SCN11A*; Table 1) encoding the α-subunit isoforms of the VGSC and four genes encoding the β-subunits (*SCN1B–SCN4B*; Table 2) have been identified in the human genome. Mutations in any of these genes can affect the structure of the channel and, thus, its biophysical properties leading to the development of “*channelopathies*” (Tables 1 and 2). All these conditions are associated with autosomal dominant inheritance and *de novo* mutations have been identified. These channelopathies can be divided in four disease groups depending on the predominant organ involved (George, 2005):

1. *Brain sodium channelopathies*, which include mutations in *SCN1A, SCN2A, SCN3A*, and some mutations in *SCN8A* observed in cases of familial human ataxia and in mice models of ataxia and end-plate diseases (these genes encode the channels Na_V_1.1, Na_V_1.2, β1-subunit, and Na_V_1.6 respectively). *SCN1A*, *SCN2A*, and *SCN3A* gene mutations may give rise to epilepsy and epileptic/convulsive disorders.
2. *Skeletal muscle sodium channelopathies*. This group involves mutations in the *SCN4A*, the gene that encodes the Na_V_1.4 isoform (skeletal muscle specific isoform). *SCN4A* gene mutations are associated with myotonia, myasthenia syndromes, and paralysis.
3. *Cardiac sodium channelopathies*, which involve mutations in *SCN5A* (the gene that encodes Na_V_1.5, which is predominantly found in cardiac muscle) and *SCN10A* (the gene that encodes Na_V_1.8, which has been recently identified in the heart (Facer et al., 2011; Verkerk et al., 2012; Yang et al., 2012) and has been associated in genome-wide association studies (GWAS) with alterations in the ventricular conduction (Chambers et al., 2010; Sotoodehnia et al., 2010).
4. *Peripheral nerve sodium channelopathies*, which include mutations in *SCN9A* (Na_V_1.7), *SCN10A* (Na_V_1.8), and *SCN11A* (Na_V_1.9). Mutations in these genes have been associated with peripheral pain syndromes (hyperalgesic syndrome) including neuropathic and inflammatory pain.

### Brain sodium channelopathies

The most commonly affected gene is *SCN1A*^1^^,^^2^. Functional studies of *SCN1A* missense epileptogenic mutations *in vitro* have been controversial but several results are consistent with loss of function (hypoexcitability) mutations (Ragsdale, 2008; Mantegazza et al., 2010) and data obtained with animal models have confirmed this (Tang et al., 2009; Martin et al., 2010). Data obtained with a mouse model of Dravet syndrome expressing a truncated Na_v_1.1 showed that loss of function of this VGSC causes reduced sodium current and excitability in GABAergic neurons, consistent with reduced GABAergic inhibition (Yu et al., 2006). Na_v_1.1 missense mutations can induce loss of function because of folding defects and these mutants can be rescued by molecular interactions with co-expressed proteins and drugs; this may be one of the causes of the phenotypic variability in GEFS+ and may be exploited for therapeutic potential (Escayg et al., 2000; Meisler and Kearney, 2005; Rusconi et al., 2007, 2009; Catterall et al., 2008). Epilepsy has also been related with *SCN1A* mutations that alter channel inactivation, resulting in persistent inward sodium current [gain-of-function (hyperexcitability) mutations; Lossin et al., 2002]. The above paragraph describes functional studies with Na_v_1.1 mutants that yield a wide range of biophysical phenotypes from loss of function to gain-of-function. At first sight this seems to be contradictory. Therefore, questions arise as to how mutations with such diverse functional effects can be associated with the same epileptic syndrome or disease. To better understand this, it is critical to remember that *SCN1A* is widely expressed in most neurons in the brain. It has also been previously reported that a single sodium channel mutation can produce hyper- or hypoexcitability in different types of neurons (Rush et al., 2006). Therefore, the net effect of the *SCN1A* mutations on the brain excitability will not only depend on the type of neuron where the mutant channel is expressed but also on the electrical balance between all the ionic currents that contribute to the neuronal AP and the mutant currents. There also might be several additional pathogenic mechanisms involved in the production of epilepsy that are not completely understood yet but still make a significant contribution to the production of the disease.

SMEI is a rare disorder characterized by generalized tonic, clonic, or tonic-clonic seizures that are initially induced by fever and begin during the first year of life. Typically, children with Dravet syndrome [or myoclonic epilepsy of infancy (SMEI); Claes et al., 2001] carry *de novo* mutations not inherited from their parents. Later, patients also manifest other seizure types, including absence, myoclonic, and simple and complex partial seizures. Psychomotor development delay is observed around the second year of life. SMEI is considered to be the most severe phenotype within the spectrum of generalized epilepsies with febrile seizures-plus. Because of this, genetic screening for *SCN1A* has become the diagnostic tool for children with early-onset seizures. More than half of the SMEI mutations cause loss of function as a result of stop codons or deletions, leading to decreased levels of functional sodium channels.

*SCN1A* mutations have also been associated with three other epileptic disorders: intractable childhood epilepsy with generalized tonic-clonic (ICEGTC) seizures, familial febrile convulsions type 3A (FEB3A), and familial hemiplegic migraine type 3 (FHM3). ICEGTC has been included in the Dravet syndrome (Mullen and Scheffer, 2009). A mutation causing simple familial febrile convulsions has been studied by Mantegazza et al. (2005). Familial hemiplegic migraine type 3 is a distinct disease caused by missense mutations of Na_v_1. Here again the functional effects are controversial, but gain-of-function effects have been observed and this is consistent with the pathogenic mechanism of migraines with aura (Cestele et al., 2008; Kahlig et al., 2008).

Missense mutations of *SCN2A* were also identified in a small percentage of GEFS+ patients and mainly in patients with benign familial neonatal-infantile seizures (BFNIS), a syndrome of mild seizures that remit during the first year of life without neurologic sequelae. BFNIS mutations produced abnormalities in the sodium channels that led to a reduced channel activity (loss of function; Misra et al., 2008). Other groups have reported mutations in *SCN2A* that result in a gain-of-function, consistently with the role of Na_v_1.2 in excitatory cortical neurons (Scalmani et al., 2006; Liao et al., 2010).

The first *SCN3A* mutation (K353Q) was identified in a patient with partial epilepsy resistant to antiepileptic drugs (Holland et al., 2008). Even though the missense mutation described caused an increase in late current, the pathogenic role of mutated Na_v_1.3 is still debated. In mouse models, mutations in *SCN8A* lead to ataxia and end-plate disease. These conditions can be reproduced by conditional knockout of *SCN8A* in cerebellar Purkinje and granule cell neurons (Levin et al., 2006). In rare cases of human familial ataxia, one frame-shift mutation has been identified in *SCN8A* that truncates the protein at the DIV and lead to loss of channel function (Vicart et al., 2005; Trudeau et al., 2006) (For more detailed information about mutations of brain VGSC see Catterall, 2010; Mantegazza et al., 2010).

### Skeletal muscle sodium channelopathies

The second group of channelopathies includes mutations in the *SCN4A* gene, which is expressed in skeletal muscle. These skeletal muscle channelopathies (sodium channel myotonic disorders) are part of a group of diseases called non-dystrophic myotonias (Matheus et al., 2010). The clinical disorders can be split between two groups based on the presence or absence of episodic weakness: paramyotonia congenita (characterized by a marked worsening of myotonia by cold and by the presence of clear episodes of weakness), and sodium channel myotonia (notable for the absence of episodic weakness but still have cold sensitivity). The latter group includes all the pure myotonic phenotypes, including the potassium-aggravated myotonias (Fournier et al., 2004).

Causative mutations in the *SCN4A* gene result in a gain of sodium channel function that may show marked temperature dependence. Almost all mutations (over 40) that have been described are missense mutations with an exception of a three base pair deletion (Michel et al., 2007). Exons 22 and 24 are the main exons involved in paramyotonia congenita, including mutations T1313M, V1589M, and mutations at the R1448 and G1306 position (Vicart et al., 2005; Matheus et al., 2008). Lerche et al. (1993) reported a group of heterozygous mutations at the G1306 position of the *SCN4A* gene. Electrophysiological studies on patient muscle samples showed slower sodium fast channel inactivation and an increase in late channel opening resulting in a steady-state inward current, sustained muscle depolarization, and muscle fiber hyperexcitability. These findings suggest that *SCN4A* residue 1306 is important for sodium channel inactivation (Lerche et al., 1993).

Finally, numerous mutations in *SCN4A* gene have also been related to hypokalemic periodic paralysis. This is a muscle disease characterized by episodes of extreme muscle weakness, and it usually begins in infancy or early childhood. Most often, these episodes involve a temporary inability to move muscles in the arms and legs. Sokolov et al. (2007) reported three mutations in gating-charge-carrying arginine residues in an S4 segment that cause hypokalemic periodic paralysis. The mutations induce a hyperpolarization-activated cationic leak through the voltage sensor of the skeletal muscle Na_V_1.4 channel, consistent with a gain-of-function. This “gating pore current” is active at the resting membrane potential and closed by depolarizations that activate the voltage sensor. The results reported by these authors showed a clear correlation between mutations that cause gating pore current and hypokalemic periodic paralysis.

### Cardiac sodium channelopathies

Na_v_1.5, encoded by *SCN5A*, conducts the inward sodium current (*I*_Na_) that initiates the cardiac AP. *SCN5A*-mediated late sodium current also influences repolarization and refractoriness. Mutations in the *SCN5A* gene result in alterations in the function of the α-subunit of the cardiac isoform Na_v_1.5 channel that have been associated with several inherited arrhythmia syndromes. The main entities related to *SCN5A* mutations include an autosomal dominant form of the LQTS (LQT3; Wang et al., 1995), BS (Probst et al., 2003), progressive cardiac conduction disease (CCD; Scott et al., 1999), sinus node dysfunction (SND; Benson et al., 2003), AF (Olson et al., 2005; Darbar et al., 2008), atrial standstill (Tan, 2006; Remme et al., 2008), and dilated cardiomyopathy (DCM; McNair et al., 2004). Most of these diseases are associated with an increased risk of sudden cardiac death (SCD). The malfunction of the β-subunits (β1 through β4) as well as some of the protein partners that interact with Na_v_1.5 α-subunit (like caveolin-3 and α-1 syntrophin) have been recently associated with diseases that resemble these arrhythmia phenotypes (Vatta et al., 2006; Cronk et al., 2007; Wu et al., 2008; Watanabe et al., 2009).

Long QT syndrome is characterized by a cardiac repolarization abnormality, with a prolonged QT interval duration observed on 12-lead ECG and vulnerability to a polymorphic ventricular tachycardia called Torsade de Pointes. About 5–10% of LQTS cases are related to mutations in *SCN5A* (LQT3) or the genes that encode the ChiPs. Mutations in *SCN5A* compromise the II–IV linker and disrupt fast inactivation, allowing repeated reopening of the channel during sustained depolarization. As a consequence, a small persistent sodium current is evoked during the AP plateau. This excessive inward current (gain-of-function) delays the repolarization of the cell, prolonging AP duration, and increasing the risk for ventricular arrhythmias.

Brugada Syndrome is a genetic disease that has been associated with ventricular fibrillation and SCD in young people. Approximately 20% of these patients have mutations in the *SCN5A* gene. More than 200 mutations have been associated with this disease (Kapplinger et al., 2010). In contrast to LQTS3 mutations, *SCN5A* mutations related to BS result in a loss of function of the channel. This can be produced by a confluence of different mechanisms, such as trafficking defects, generation of defective or truncated proteins, faster channel inactivation, shift of voltage-dependence inactivation toward a more depolarized membrane potential, or even slow recovery from inactivation. The electrical consequence of this is the presence of a slower conduction substrate. BS has also been associated with mutations in the genes that encode β1- (*SCN1B*, BS type 5) and β3-subunits (*SCN3B*, BS type 7; Abriel, 2010) of the cardiac sodium channel.

Genetic mutations in *SCN5A* specific only to AF have recently been described. Recently, Li et al. (2009) identified a novel coding variant, K1493R, which altered a highly conserved residue in the DIII–IV linker and was located six amino acids downstream from the fast inactivation motif of sodium channels. Biophysical studies of K1493R in tsA-201 cells demonstrated a significant positive shift in voltage-dependence of inactivation and a large ramp current near resting membrane potential, indicating a gain-of-function. Enhanced cellular excitability was observed in transfected HL-1 atrial cardiomyocytes, including spontaneous AP depolarizations and a lower threshold for AP firing. These novel biophysical observations provide molecular evidence linking cellular “hyperexcitability” as a mechanism inducing vulnerability to AF.

Other pathologies related to mutations in *SCN5A* include progressive familial heart block type 1A (PFHB1A), sick sinus syndrome type 1 (SSS1), sudden infant death syndrome (SIDS), familial atrial standstill, and DCM. For a more detailed review on *SCN5A* channelopathies see Zimmer and Surber (2008), and Wilde and Brugada (2011).

In addition to *SCN5A* mutations, variants in *SCN10A* (the gene that encodes Na_V_1.8) can also lead to alterations in the cardiac rhythm. Na_V_1.8 has only recently been identified in the heart (Facer et al., 2011; Verkerk et al., 2012) and GWAS have identified common genetic variants in this gene that modulate ventricular conduction (Chambers et al., 2010; Sotoodehnia et al., 2010).

### Peripheral nerve sodium channelopathies

Lampert et al. and Theile and Cummins, recently published extensive reviews on the role of sodium channels in chronic and neuropathic pain syndromes (Lampert et al., 2010; Theile and Cummins, 2011). Neuropathic pain is defined as “pain caused by a lesion or disease of the somatosensory nervous system,” and can be divided into central and peripheral neuropathic pain. Typical examples of neuropathic pain include post-herpetic neuralgia, painful diabetic neuropathy, phantom limb pain, and spinal cord injury pain. The fundamental mechanism involved in the production of neuropathic pain is an increase in nerve excitability (and thus changes in VGSC properties), generally manifested in impulses generated ectopically or with minimal stimulation. Nerve injury (classically associated with neuropathic pain) can result in changes in sodium channel trafficking, gene expression, and/or channel kinetics, all of which contribute to neuronal membrane remodeling and hyperexcitability associated with neuropathic pain (Devor, 2006). VGSC Na_v_1.7, Na_v_1.8, and Na_v_1.9 have been particularly identified in the PNS (peripheral neurons and DRG neurons) and seem to have a central role in the genesis of neuropathic pain. Thus, these channels are the new targets for analgesia in peripheral neuropathy pain syndromes. In particular, Na_v_1.7 is considered to be one of the main mediators of peripheral pain. It has been recently reported that Na_v_1.8 sodium channel is part of the molecular machinery involved in mechanotransduction of joint pain and other pain syndromes (Schuelert and McDougall, 2012). On the other hand, the role of Na_v_1.3 in diseased states is still controversial.

Recent human association studies have directly linked *SCN9A*, the gene that encodes Na_v_1.7, to three human pain disorders: dominantly inherited gain-of-function mutations in inherited erythromelalgia (IEM; nine mutations), paroxysmal extreme pain disorder (PEPD; eight mutations), and recessively inherited loss-of-function mutations in Na_v_1.7-related congenital insensitivity to pain (CIP; fourteen mutations) (Dib-hajj et al., 2009).

Inherited erythromelalgia (IEM) is a chronic neuropathic pain syndrome that is characterized by excruciating painful attacks in the extremities that begin in childhood and progress over life. A shift to voltage-dependent activation toward more negative potentials seems to be a common factor in all the mutations of *SCN9A* that lead to this disease. This leftward shift of activation can lead to a hyperexcitability state (gain-of-function mutations). Many mutations also delay inactivation, and therefore, larger currents result from slow depolarizing stimuli (“*ramp currents*”).

Paroxysmal extreme pain disorder (PEPD), previously referred to as familial rectal pain (Fertleman et al., 2006), is characterized by severe pain accompanied by flushing which are induced by bowel movements or probing of the perianal areas, and are sometimes accompanied by tonic non-epileptic seizures, syncope, bradycardia, and occasionally asystole.

Congenital insensitivity to pain (CIP) is characterized by complete absence of pain perception in patients with non-functional Na_v_1.7. These patients also exhibit partial anosmia. In this case, the mutations in *SCN9A* identified introduce a stop codon leading to the production of truncated proteins that are non-functional. For further details on the mutations related to each of these diseases see Lampert et al. (2010).

## Summary

Voltage-gated sodium channels are widely distributed in excitable and non-excitable cells, and play a critical role in electrical activation in the body. VGSC constitute macromolecular complexes, in which their function relies on both the specific structure of the channel protein (α- and β-subunits) as well as their protein partners (ChiPs). Since VGSC occur predominantly in the central and PNS, and striated (skeletal and cardiac) muscles, mutations in genes encoding VGSC and ChiPs will culminate in diseases named “*channelopathies*” that can be grouped into four main categories: epileptic syndromes, skeletal myopathies, cardiac arrhythmias, and neuropathies (with pain-related syndromes). Pathologic conditions can also arise from the up regulation of the VGSC, as for example in highly aggressive prostate (Na_v_1.7) and breast (Na_v_1.5) metastatic carcinomas, An improved understanding of the critical role of the molecular composition of ion channel complexes, the influence of protein partners, and the specific cellular domains underlying protein interactions, are essential for the development of new therapies to treat channelopathies associated with VGSC.

## Conflict of Interest Statement

The authors declare that the research was conducted in the absence of any commercial or financial relationships that could be construed as a potential conflict of interest.

## Footnotes

^1^http://www.molgen.ua.ac.be/SCN1AMutations

^2^http://www.scn1a.info/
